# Limited joint mobility syndrome: a striking clinical sign not to be missed

**DOI:** 10.11604/pamj.2025.50.106.47598

**Published:** 2025-04-22

**Authors:** Naushad Abid, Hamzah Naushad Siddiqui

**Affiliations:** 1 Department of Internal Medicine, College of Medicine, King Faisal University, Hofuf, Saudi Arabia,; 2 Dow International Medical College, Karachi, Pakistan

**Keywords:** Limited joint mobility syndrome, diabetes, microvascular complications

## Image in medicine

Limited joint mobility syndrome (LJMS) of the hands and fingers, also called cheiroarthropathy, is characterized by painless hand and finger stiffness. It is an under-recognized condition often associated with diabetes-related complications. We present a case of a 65-year-old male with a 20-year history of type 2 diabetes who came to a general practitioner four years ago with progressive hand stiffness. He was prescribed only analgesics. Two years later, he began to experience shortness of breath and lower limb pitting edema and was ultimately diagnosed with ischemic cardiomyopathy. Examination revealed features of heart failure, an inability to approximate the palmar surfaces of the hands referred to as the “prayer sign,” and tightened skin on the dorsal and palmar aspects of the hands. Laboratory tests showed elevated B-type natriuretic peptide (BNP) and proteinuria. We diagnosed limited joint mobility syndrome secondary to long-standing diabetes complicated by microvascular issues. We believe our patient had subclinical atherosclerosis at the time of his initial presentation four years ago, and timely recognition and evaluation could have prevented or delayed the onset of later complications. A cross-sectional study also found high carotid intimal-media thickness (IMT) and plaque scores in patients with LJMS of the hands, highlighting the importance of identifying LJMS. Timely screening of the musculoskeletal system in diabetes may delay or prevent further complications.

**Figure 1:**
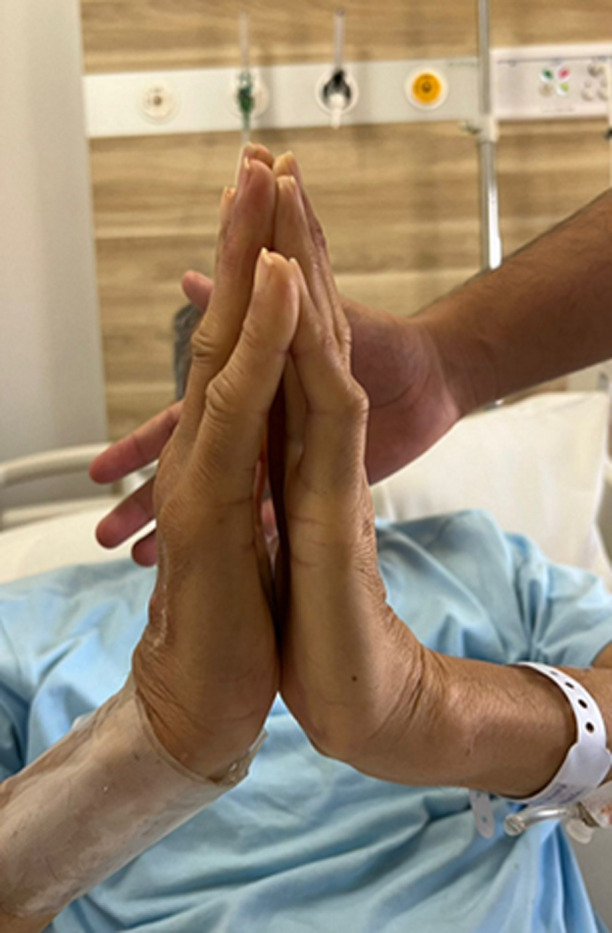
prayer sign in limited joint mobility syndrome

